# HDX-MS analysis of ribosome-nascent chain complexes to study protein biogenesis at the peptide level

**DOI:** 10.1038/s41596-025-01279-w

**Published:** 2026-01-12

**Authors:** Alžběta Roeselová, Aleksandra Pajak, Thomas E. Wales, Grant A. Pellowe, Svend Kjær, John R. Engen, David Balchin

**Affiliations:** 1 Protein Biogenesis Laboratory, The Francis Crick Institute, London, UK; 2 Department of Chemistry & Chemical Biology, Northeastern University, Boston, MA, USA; 3 Structural Biology Science Technology Platform, The Francis Crick Institute, London, UK

## Abstract

Nascent proteins begin to fold during their synthesis, while still attached to the ribosome. The dynamic nature of ribosome-nascent chain complexes (RNCs) poses a significant challenge for conventional structural biology approaches, limiting our understanding of dynamic cotranslational events. Hydrogen-deuterium exchange mass spectrometry (HDX-MS) is a powerful technique for studying the conformational equilibria and refolding of full-length proteins, label-free and with peptide resolution. However, the large size of the ribosome and the need for stable, highly homogeneous samples have hindered the application of HDX-MS to RNCs. Here, we present a strategy for analysing conformational dynamics and interactors of *E. coli* RNCs using HDX-MS. High-quality RNCs are obtained through the gentle lysis of high-density cultures expressing uniformly stalled ribosomes, followed by ultracentrifugation and tag-based affinity purification. Peptide-resolution information on protein conformational dynamics is obtained by pulse deuterium labelling, quenching with an RNA-compatible low pH buffer, and offline digestion with pepsin. Extensive data analysis with use of specific internal controls allows for the confident assignment of mass spectra to specific peptides, ensuring good coverage of the nascent chain and ribosomal proteins. This method provides a valuable complement to existing structural techniques such as cryo-electron microscopy and nuclear magnetic resonance, and enables detailed characterization of large, partially structured nascent chains and their interactions with the ribosomal proteins and molecular chaperones. The protocol takes 1-3 months, from sample preparation and data acquisition to data analysis, and requires standard expertise in cloning and protein purification and intermediate expertise in HDX-MS.

## Introduction

Proteins are synthesized by the ribosome during mRNA translation. The ribosome is made up of multiple rRNA molecules and over 50 proteins, which together function to decode mRNA and catalyse peptide bond formation to add amino acids to growing nascent chains (NCs) ^1^. The synthesised chains must subsequently fold into a specific conformation necessary for their cellular function. Since the speed of protein folding ^2^ is faster than speed of mRNA translation ^3^, most nascent chains initiate folding cotranslationally on the ribosome (reviewed extensively in previous literature ^4–8^).

Cotranslational folding during *de novo* synthesis on the ribosome occurs in a very different context than (re)folding of full-length proteins post-translation. During translation, the ribosome exit tunnel sequesters ~30-40 residues of the NC in an extended conformation, but can permit formation of short α-helices inside the tunnel ^9–15^ or even small tertiary structures in the vestibule where the exit tunnel widens ^16–21^. As the NC chain emerges from the ribosome the strict spatial constraints of the tunnel are relieved, but the ribosome continues to influence NC folding. Ribosome-proximal sites of the NC can directly interact with the charged ribosomal surface ^22–25^, which can influence the folding kinetics and thermodynamics of nascent polypeptides even outside the exit tunnel. In general, folded nascent chains have been shown to reduce in thermodynamic stability on the ribosome ^26–31^. This has been linked to direct ribosome:NC interactions or to charge repulsion and space constraints near the exit tunnel, which all diminish with increasing distance from the ribosome ^32–36^. Moreover, since the ribosome synthesizes proteins by addition of residues at the C-terminus, the N-terminal segments can interact with molecular chaperones and begin to fold independently of the context of the not-yet synthesized C-terminal sequence. The gradual emergence of the nascent chain can prevent non-productive long-range interactions, promoting segmental folding ^37,38^.

Cotranslational folding pathways can differ from refolding of full-length proteins ^19,30,39,40^, and interfering with NC:ribosome interactions and the order of domain synthesis can lead to misfolding and aggregation of newly synthesised proteins ^22,41,42^. However, only a limited number of studies to date provide a detailed molecular explanation for these observations. The complexity of the ribosome, temporal dynamics of protein synthesis, conformational dynamics of folding intermediates, and the occurrence of ribosome:NC and chaperone:NC interactions, all present a challenge to characterising cotranslational folding. Hence, the field relies on an array of methods ^43^, from *in vivo* based assays reflecting the complete context of protein synthesis to biophysical approaches offering detailed information about folding states and interaction surfaces.

To obtain detailed information about structural dynamics of RNCs and chaperone-RNC complexes, most studies rely on isolating stalled translation intermediates in the form of ribosome nascent chain complexes (RNCs). RNCs can be captured by translating mRNAs without a *Stop* codon ^44^, but more stable stalling relies on specific stalling sequences ^45^ such as those derived from *E. coli* SecM ^46,47^. Stalled RNCs can be expressed *in vitro* or *in vivo* and purified to low µM concentrations, making them suitable for many biophysical methods ^48–53^. RNCs have been studied using NMR, cryoEM, limited proteolysis, optical tweezers, fluorescence anisotropy depolarization, FRET, force profile analysis and other methods ^43^. While powerful, these approaches are often limited in one or more respects. They can be poorly suited to large multi-domain proteins, rely on bespoke optimisation for specific NCs, do not report local folding information, require extrinsic labels, or are confounded by chaperone binding. To contribute to the range of approaches for characterising protein maturation on the ribosome, we have recently developed a protocol based on hydrogen/deuterium exchange mass spectrometry (HDX-MS) analysis of stalled RNCs. We have used this protocol to study the folding and chaperone interactions of both single- and multi-domain NCs at peptide resolution ^23–25,54^.

HDX-MS leverages the fact that main chain amide hydrogens in a D_2_O-containing solvent exchange with deuterium, with the exchange rate influenced by backbone solvent accessibility and hydrogen bonding ^55^. Given that protein folding depends on hydrogen bond-stabilized secondary structures, HDX-MS is particularly suited for probing the conformational dynamics and folding states of translation intermediates at peptide resolution ^56^. Mass changes <1 Da (a single exchanged amide H) can easily be detected, providing a stringent measure of folding status and sensitive detection of conformational changes in ribosomal proteins or NC interactors such as chaperones. Furthermore, HDX-MS has the potential to provide insights into interaction sites between the nascent chain, the ribosome, and any RNC interactors. While HDX-MS has been used to study refolding of full-length proteins with and without chaperones ^57–59^, the complexity and instability of ribosome-nascent chain samples has hindered the application of HDX-MS to RNCs. Recent technical improvements in sample preparation, chromatography, instrumentation and data analysis have now made it feasible to overcome these challenges and adapt HDX-MS workflows for studies of large complexes.

### Overview of the procedure

**Figure 1:**
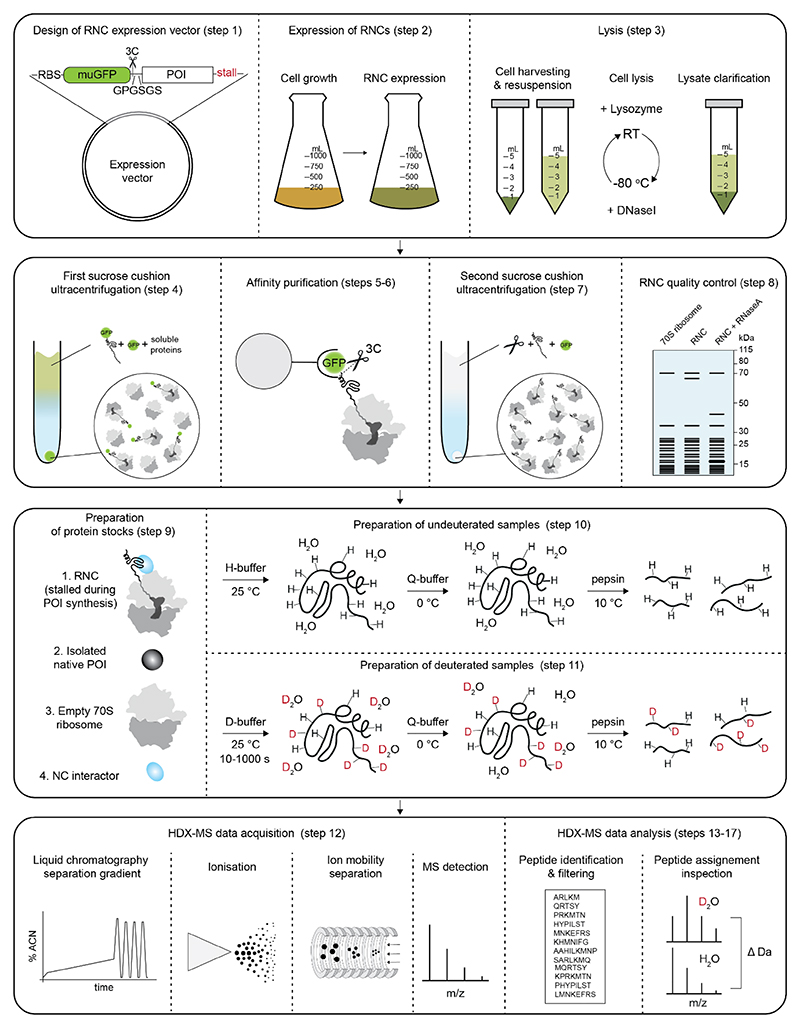
Schematic summarising the steps involved in analysis of RNCs by HDX-MS. The steps include expression and purification of stalled RNCs and HDX-MS sample preparation, followed by HDX-MS data acquisition and analysis.

Here we present a protocol to express and purify stable and homogeneous RNCs, and for subsequent analysis by HDX-MS (Fig. 1). Briefly, ribosome stalling during nascent chain synthesis is stabilised by a previously optimized 10-residue arrest peptide encoded at the C-terminus of the nascent chain ^46,60^, positioned deep inside the ribosome tunnel to avoid interfering with nascent chain folding ^22,61^ (step 1). Expression of RNCs is achieved by growth of high-density *E. coli* cultures in auto-induction media ^62^ (step 2). RNC-expressing cells are harvested and lysed using lysozyme treatment and freeze-thawing to preserve translation stalling ^50^ (step 3). Subsequent purification combines sucrose cushion ultracentrifugation (steps 4 and 7) with affinity purification (steps 5 and 6) using a cleavable N-terminal tag to isolate nascent chain-occupied ribosomes. SDS-PAGE is used to verify stalling and sample purity (step 8).

Purified RNCs, together with necessary control samples (step 9), are subsequently used to prepare undeuterated (step 10) and deuterated (step 11) samples for HDX-MS. Protease digestion results in peptic peptides which are separated using a long liquid chromatography (LC) gradient followed by ion mobility (IM) prior to mass spectral acquisition (step 12). Extensive separation of peptides during LC and IM reduces mass spectral overlap enabling peptide identification (step 13) through unambiguous spectra assignment ^63^ (steps 14-16). Positive and negative control samples are included to identify misassignments and ensure internal quality control (step 15). Finally. peptide-specific mass differences between deuterated and undeuterated conditions are calculated and used to characterise NC conformations and identify interaction sites between NCs, ribosomal proteins and RNC-interacting proteins (step 17).

This protocol has been successfully employed in published studies to elucidate chaperone-assisted cotranslational protein folding of a single-^25^ and multidomain protein ^24,54^. Our workflow has been effective in studying nascent chains with diverse characteristics, including variations in length, domain number, charge, and conformational flexibility. This protocol can benefit researchers in structural and molecular biology, particularly those studying protein synthesis, co-translational folding, nascent chain interactors and other ribosome-associated processes. Additionally, scientists in other fields may find this protocol valuable, as it outlines a strategy for studying large, flexible, nucleic acid-rich complexes by HDX-MS which provides information complementary to other structural methods such as cryoEM.

### Advantages and limitations

This protocol describes a versatile approach to produce high quantities of pure, homogenous and stable *E. coli* RNCs within 48 hours without relying on liquid chromatography systems. It can be easily adapted to a variety of NCs and allows customisation of the purification buffers, affinity tag and expression conditions. Moreover, the method can be easily scaled up and allows purification of multiple RNCs in parallel. Due to the reliance on the SecM stalling sequence, this protocol is only suitable for expression in prokaryotes. Other stalling sequences can be used to isolate eukaryotic RNCs ^23,64^.

Our protocol also presents a strategy to study RNCs by HDX-MS. Since it is sensitive to hydrogen bond formation and solvent accessibility, HDX-MS is a powerful tool to detect changes in nascent chain conformation and interactions. HDX-MS offers several advantages over existing biophysical techniques used to study RNCs, detailed in previous literature ^43,48,49^. It is not limited by the size or conformational dynamics of the nascent chain and does not require high sample concentrations. It can be used to study NCs of various length, domain number or charge, also when in complex with different interactors including proteins, peptides, small molecules or nucleic acids. This flexibility makes HDX-MS particularly complementary to NMR-studies which are best-suited to small and conformationally dynamic proteins, and cryo-EM which cannot resolve dynamic elements of the nascent chain and its interacting partners. Additionally, HDX-MS provides peptide-resolution information for the entire nascent chain, ribosomal proteins, and any RNC-interacting proteins or peptides in a single experiment. Moreover, the approach does not rely on artificial labels, which can affect protein folding and disrupt interaction surfaces.

An inherent limitation of HDX-MS experiments is that detection of increased deuterium uptake indicates a change in either hydrogen bond stability or solvent accessibility. This can result from either conformational changes or direct binding, making it challenging to unambiguously interpret partial protection from exchange. Hence, where possible, HDX-MS should be supported by appropriate controls as well as data from orthogonal approaches. Secondly, due to the complexity of the RNC samples, detection of peptides covering the NC is more challenging than in a typical HDX-MS experiment focused on an isolated protein. This can limit the resolution of the method, which is determined by coverage as well as length and overlap of peptides. Moreover, the complexity of the samples requires a more involved data analysis which can take multiple weeks. Lastly, since proteomics approaches cannot differentiate between identical peptides from different origins, this protocol may be limited when studying homooligomers or proteins with repetitive sequences.

## Experimental design

### Stage 1: Generation of stalled RNCs

Ribosome stalling can be induced site-specifically by designing expression templates encoding short arrest peptides at the C-terminus of the expressed nascent chain (step 1). Commonly used arrest peptides include sequences derived from an *E. coli* protein SecM ^50^. Upon translation, the SecM sequence interacts with the ribosome exit tunnel, resulting in an NC geometry incompatible with further nascent chain elongation ^61,65^. Because stalling induced by wild-type SecM is disrupted by folding of the upstream NC ^66–68^, this protocol utilises a short SecM variant optimised for stall stability (SecMStr - WWWPRIRGPP) ^46,60^. The presence of SecMStr on the C-terminus of a NC is not expected to affect NC folding, as SecMStr is only 10 residues and occupies the 70S ribosome exit tunnel prior to the L22/L4 constriction point ^22^ believed to unfold any pre-formed secondary structure ^9,14^.

To obtain large quantities of purified material, RNCs are generated by NC expression from recombinant genes under the control of a T7 promoter in BL21(DE3) cells using ZYM-5052 auto-induction in high density shaking cultures ^62^ (step 2). Auto-induction expression cultures can be inoculated by single colonies and then grown for overnight (16-18 hours) at 37 °C, without the need for starter cultures or monitoring optical density. IPTG-based induction can also be used for RNC expression but is less convenient and might require larger culture volumes for similar yields.

### Stage 2: Purification of stalled RNCs

**Figure 2:**
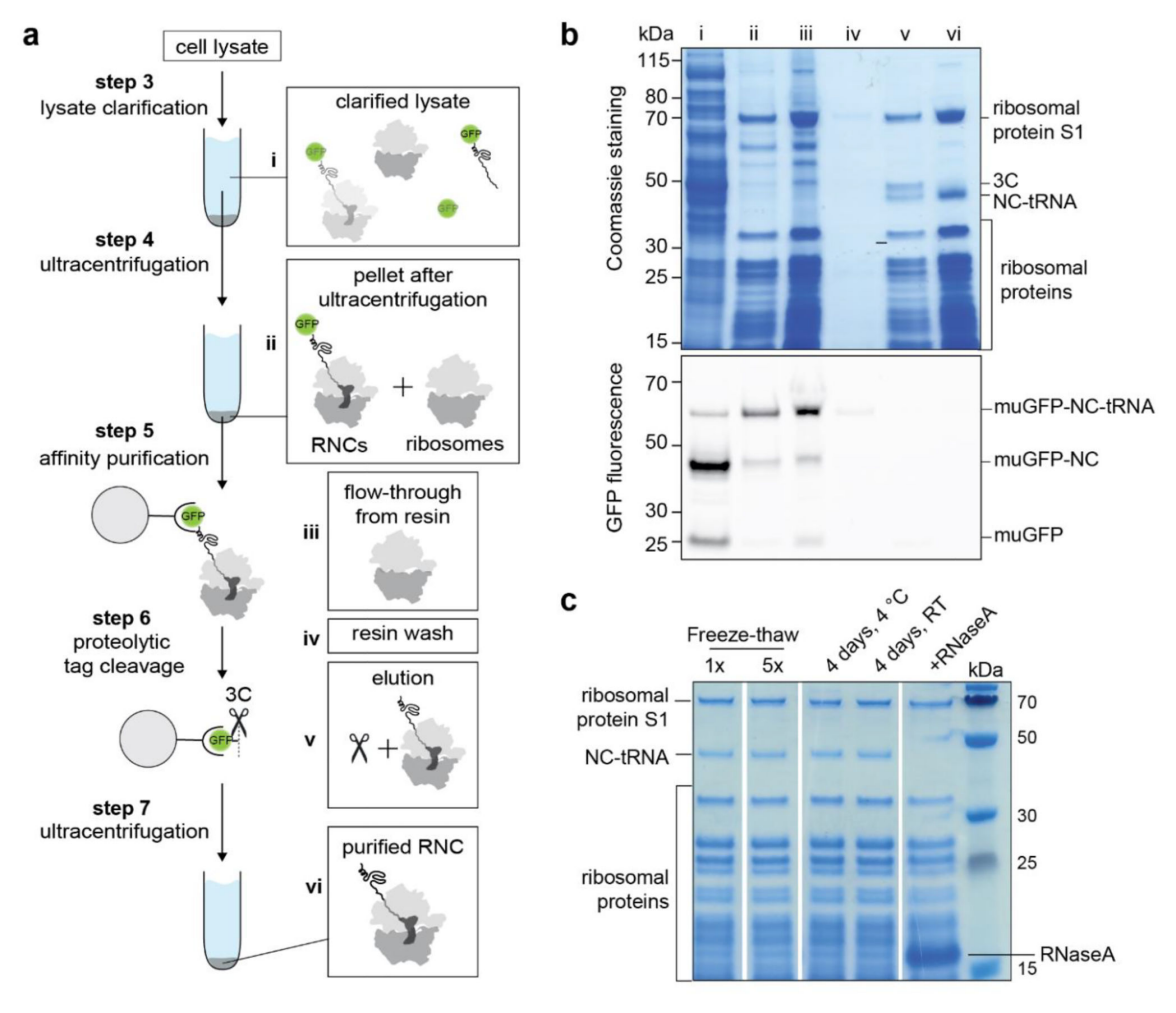
Purification and quality control of RNCs (**a**) Schematic of the RNC purification protocol (steps 3-7 in Fig. 1). The cell lysate from *E. coli* overexpressing stalling constructs is first clarified by centrifugation (step 3, i) and applied to a sucrose cushion to isolate the ribosomal fraction (ii) during ultracentrifugation (step 4). RNCs are then separated from empty ribosomes (iii, iv) in an affinity purification (step 5), utilising the muGFP-tag at the N-terminus of the NC. Next, the NC (v) is specifically eluted via tag-cleavage using HRV 3C protease (step 6). Finally, the elution is subjected to a second sucrose cushion ultracentrifugation (step 7), to yield the purified RNC (vi) cleared of 3C protease and any other small impurities. (**b**) SDS-PAGE gel stained with Coomassie (top) or scanned for GFP fluorescence prior to staining (bottom). The gel follows the purification of an *E. coli* DHFR RNC. The specific stages of the purification correspond to the schematic in (a) – clarified cell lysate (i), pellet after 1^st^ ultracentrifugation (ii), flow-through from affinity purification (iii), resin wash (iv), elution form affinity purification (v), and pellet after 2^nd^ ultracentrifugation (vi). Bands corresponding to ribosomal proteins, RNaseA, muGFP, and NCs with or without N-terminal muGFP-tag and C-terminally linked tRNA are indicated. The presence of tRNA on the NC indicates that the NC has successfully been stalled on the ribosome. *This figure was taken from published literature* ^25^. (**c**) The stability of a purified DHFR RNCs monitored by the integrity of the NC-tRNA band on a Coomassie-stained SDS-PAGE. Conditions included repeated cycles of freezing in liquid N_2_ followed by thawing on ice (freeze-thaw), or incubation for 4 days at 4 °C or 25 °C. The last lane contains a degraded RNC following a treatment with 50 µg/mL RNaseA in the presence of 50 mM EDTA. The tRNA-linked nascent chain (NC-tRNA) migrates ~20 kDa higher than NC without a tRNA. All lanes belong to the same gel, but have been rearranged. *This figure was taken from published literature* ^25^.

Harvested cells expressing stalled RNCs must be lysed without disrupting RNA or native protein:protein interactions, precluding the use of sonication. For smaller volumes, cells can be gently lysed by a combination of lysozyme treatment and freeze-thaw cycles ^50^ (step 3). For large scale expression, lysis can also be achieved by French press ^69^ or cryogenic milling ^51^. The stalled RNCs will only be a minor species in the cell lysate (Fig. 2a,b), which contains not only all other soluble proteins in the cells but also the overexpressed nascent chains which did not successfully stall. To enrich for the stalled RNCs, the crude lysate is first clarified by centrifugation to remove insoluble species, then the soluble fraction is subjected to sucrose cushion ultracentrifugation, which selectively pellets the heavy ribosomes away from smaller proteins (step 4).

An NC-specific affinity purification step is performed next (step 5), to purify occupied RNCs away from other 70S ribosomes isolated from the cell lysate. In this protocol we used an N-terminal muGFP tag (monomeric ultrastable GFP) ^70^ which is highly efficiently captured by a designed ankyrin repeat protein (DARPin) ^71^. The tag also allowed us to easily follow the NC throughout the purification process (Fig. 2a,b), by simply detecting muGFP fluorescence in solution or following SDS-PAGE without the need for immunoblotting. Since muGFP is a relatively large tag (~27 kDa) and is thus at risk of affecting downstream NC folding, we have since adapted the protocol to use other N-terminal tags, including 3xFLAG, Twin-Strep and Alfa ^72^ (unpublished). Unlike muGFP, these tags can be competitively eluted without cleaving the tag. Tags on NCs can be used to detect RNCs for immunoblotting, which is especially beneficial when studying short NCs which might not contain necessary epitopes. Unlike other methods which selectively label the NC (e.g. NMR, FRET, force spectroscopy), HDX-MS is sensitive to overall sample complexity. We therefore recommend purifying RNCs using high-affinity tags which can be selectively eluted. We do not recommend using a His_6x_-tag for the affinity purification step, since ribosomes have previously been reported to interact with metal-chelating resins ^53^. Uniformly occupied RNCs are also a requirement for quantitative analysis of chaperone:RNC interactions.

Following elution from the affinity resin (step 6), a second sucrose cushion ultracentrifugation step is used to concentrate purified RNCs and isolate them from any contaminating unstalled NCs and unwanted buffer supplements used for elution such as tag-cleaving proteases (step 7). After every purification, the purity of the samples, occupancy of the ribosome by the NC, integrity of the ribosomes, presence of tRNA on the NC, and stability of stalling should be confirmed using SDS-PAGE optionally coupled with immunoblotting (step 8, Fig. 2c). With the complexity of the sample containing all ribosomal proteins, SDS-PAGE may not reveal contaminating proteins. We therefore also strongly recommend additional mass spectrometry analysis as a quality control step to determine the composition of each purified RNCs the first time they are prepared.

### Stage 3: Sample preparation for HDX-MS

HDX-MS takes advantage of the fact that in a D_2_O-containing solvent, hydrogens can exchange with deuterium in solution. The exchange can be measured by mass spectrometry, as deuterium D (~2.01 Da) is heavier than protium H (~1.01 Da) ^73^. The rate of exchange differs depending on the chemical environment, with only the backbone amide hydrogens exchanging at rates measurable during a typical HDX-MS experiment ^74^. This makes HDX-MS sensitive to incorporation of deuterium at any amino acid except proline. The exact rate of HDX is affected by temperature, pH, solvent accessibility and amide hydrogen bonding. Since temperature and pH can be experimentally controlled, HDX-MS is used to specifically detect hydrogen bonding and solvent accessibility of the protein backbone.

In a typical HDX-MS workflow (steps 9-11), a protein of interest is first introduced into a D_2_O-based buffer allowing for hydrogen-deuterium exchange to take place (step 11). After incubation at a controlled temperature for a certain amount of time, typically in the seconds to hours range, the labelling reaction is quenched by lowering the temperature and adjusting the pH of the sample to ~2.5 using a low pH H_2_O-based buffer ^75^. To localise the sites of deuterium incorporation, the samples are next digested with a protease. The most common protease used for this step is pepsin, as it is stable under low pH quench conditions ^76^. In this protocol we carry out digestion “offline” (i.e. uncoupled from the LC system), to prevent LC column damage from excess RNA ^77^ and minimise carry-over between samples.

Since pepsin cleaves proteins non-specifically, it cannot be rationally predicted which peptides will result from the digestion. Hence, it is necessary to prepare samples which were not labelled by a deuterium pulse, but which were otherwise processed the same way as deuterated samples (step 10). All samples are typically prepared in technical replicates for any given deuteration time point, to increase the chance of detecting a specific peptide and to control for variability in D-uptake introduced during sample preparation and data acquisition.

### Stage 4: HDX-MS data acquisition

Following digestion by pepsin, the resulting peptides are injected into a UPLC system maintained at 0 °C to minimise back-exchange of deuterium to hydrogen (step 12). Peptides are trapped, cleaned from salts and detergents, and separated by reverse-phase liquid chromatography. Since the liquid chromatography is the slowest step in the HDX-MS workflow following deuteration, a balance needs to be established between long enough LC gradient to achieve good peptide separation in the complex sample, and short enough LC gradient to avoid excessive loss of deuterium signal due to back-exchange. Peptides eluting from the LC are ionized using electrospray ionization and further separated by their collisional cross-section and charge in an ion mobility step. The mass to charge ratio (m/z) of peptides is subsequently measured using a mass spectrometer and the MS spectra are recorded together with the corresponding LC retention time and ion mobility value. Additionally, tandem MS data are collected by introducing an additional collision gas fragmentation step.

### Stage 5: HDX-MS data analysis

First, tandem MS data collected from undeuterated samples are used to identify peptic peptides. This is done by comparing the experimental data to *in silico* simulated fragmentation data derived from a database of protein sequences present in the samples (step 13). After the identification of candidate peptides, the peptide list is filtered by preferred characteristics and the MS^1^ spectra of all samples are, when possible, assigned to peptides in the list (step 14).

Since the RNC samples contain over 50 ribosomal proteins ^1^, the NC, and possibly NC-interactors such as chaperones, they give rise to very complex MS data. In our experience misassignments occasionally occur, necessitating the use of additional control samples in the form of empty 70S ribosomes and, where possible, isolated proteins of interest to reduce risks of false assignments (step 9). Subsequently, it is necessary to manually inspect all assigned spectra and exclude any low-quality peptides, peptides that have been assigned ambiguously, or those that have been assigned in samples which are known not to contain the protein from which the peptide was derived (steps 15-16). Overlapping peptides should exhibit similar deuterium uptake and hence can be useful when identifying outliers and misassigned peptides.

The final peptide list with the corresponding undeuterated and deuterated spectra can be used to calculate the mass-to-charge ratio (m/z) centre of spectral mass (centroid) of each isotopic envelope and derive the relative deuterium uptake at different labelling times at peptide resolution (step 17). Peptides with larger differences between m/z in the labelled and unlabelled conditions represent sites which are more conformationally dynamic or solvent accessible.

## Materials

### Reagents

**CRITICAL** All reagents should be purchased at the highest possible grade to make sure they are RNase-free and do not contain impurities that would interfere with mass spectrometry data acquisition. Wear gloves when handling reagents to prevent contamination. Unless otherwise stated, listed reagents can be substituted by equivalent reagents from user’s preferred vendor.

1. NaOH **! CAUTION** NaOH is corrosive.
2. NaOD - **! CAUTION** NaOD is corrosive.
3. HCl - **! CAUTION** HCl is corrosive.
4. DCl - **! CAUTION** DCl is corrosive.
5. Orthophosphoric acid - **! CAUTION** orthophosphoric acid is corrosive.
6. NaCl
7. Na_2_SO_4_
8. Na_2_HPO_4_ x 7 H_2_O
9. NaH_2_PO_4_ x H_2_O
10. KCl
11. KOAc
12. K_2_HPO_4_
13. KH_2_PO_4_
14. MgCl_2_ x 6 H_2_O
15. Mg(OAc)_2_ x 4 H_2_O
16. MgSO_4_
17. NH_4_Cl
18. HEPES
19. Trizma base
20. TCEP HCl
21. DTT
22. β-mercaptoetahnol
23. Glucose
24. α-lactose
25. Imidazole - **! CAUTION** imidazole is corrosive.
26. Glycine
27. Tryptone
28. Bacto Yeast Extract
29. Glycerol
30. 0.5 M EDTA, pH 8.0
31. Ampicillin sodium salt
32. Chloramphenicol
33. IPTG
34. Trace metals: FeCl_3_, CaCl_2_, MnCl_2_ x 4 H_2_O, ZnSO_4_ x 7 H_2_O, CoCl_2_, CuCl_2_x2 H_2_O, NiCl_2_ x 6 H_2_O, Na_2_MoO_4_, or Na_2_SeO_3_
35. Trisodium citrate
36. Sodium cyanoborohydride - ! **CAUTION** sodium cyanoborohydride is flammable, corrosive and toxic.
37. Ethanolamine (16.5M, Merck, 411000) **! CAUTION** ethanolamine acid is corrosive.
38. Guanidine hydrochloride (ThermoScientific, 15502016)
39. Urea, ultrapure (ThermoScientific, J65769-22)
40. RNase-free sucrose (VWR, 0335)
41. Rosetta 2 (DE3) pLysS (Merck, 71403)
42. BL21(DE3) *E. coli* (NEB, C2527)
43. Expression vector encoding the His-tagged KKK_nl_gc_R7 GFP-clamp variant
44. RNC expression vector personalised by user
45. Ethanol 99.8+%
46. RNase-free water (ThermoScientific, 10977035)
47. RNase-free DNase (QIAGEN, 79254)
48. Trifluoroacetic Acid, Optima LC/MS Grade (ThermoFisher, A11650)
49. Water with 0.1% Formic Acid (v/v) Optima LC/MS Grade (ThermoFisher, LS118)
50. Acetonitrile Optima LC/MS Grade (ThermoFisher, A955)
51. Deuterium oxide (Merck, 151882)
52. Halt Protease Inhibitor Cocktail (ThermoScientific, 78437)
53. Protease-inhibitor tablet cOmplete EDTA-free (Roche, 11873580001)
54. RiboLock RNase Inhibitor (ThermoScientific, EO0384)
55. Skimmed dry milk (Marvel)
56. Lysozyme
57. GST-tagged human rhinovirus 3C protease (such as Merck, GE27-0843-01) - we used an equivalent lab-generated recombinant GST-3C protease purified at 5 mg/mL
58. RNaseA (NEB, T3018L)
59. [Glu^1^]-Fibrinopeptide B (Merck, F3261)
60. Pepsin from porcine gastric mucosa (Merck, P6887)
61. *E. coli* 70S ribosomes (NEB, P0763S)
62. Pierce NHS-Activated Agarose (ThermoScientific, 26200)
63. POROS 20 AL Aldehyde Activated Resin (ThermoScientific, 1602906)
64. Quick Coomassie Stain (Neo Biotech, NB-45-00078)
65. Bis-Tris polyacrylamide gel (ThermoScientific)
66. NuPAGE MES SDS Running Buffer (20X) (ThermoScientific, NP0002)
67. PageRuler Plus Prestained Protein Ladder (ThermoScientific, 26619)
68. NuPAGE LDS Sample Buffer (ThermoScientific, NP0008)
69. SuperSignal West Pico PLUS Chemiluminescent Substrate (ThermoScientific, 34577)
70. Tween-20 (Merck, P1379)

### Equipment

**CRITICAL** Unless otherwise stated, the listed equipment and software may be substituted with equivalents from the user’s preferred vendor.

1. Standard plastic laboratory tubes 1.5-250 mL
2. Polypropylene Protein LoBind Tubes (0.5-2 mL, Eppendorf) – **CRITICAL** these tubes are RNAse-free and are coated with MS-compatible reagents
3. General chemical glassware
4. UV-visible spectrophotometer
5. Shaking incubator for 1 L flasks
6. Shaking incubator for 1.5 mL tubes (Thermocycler)
7. Gel imaging system capable of fluorescence and chemiluminescence detection
8. pH meter
9. Ultrasonic Cell Disruptor (sonicator)
10. SDS-PAGE running equipment
11. Trans-Blot Turbo Transfer System (BioRad)
12. PVDF membrane (BioRad, 1704156)
13. Vortex mixer
-80 °C freezer
15. Liquid nitrogen
16. Autoclave
17. Centrifuge(s) capable of 1,000-16,000 g with rotors and adaptors for 1.5-250 mL tubes
18. Ultracentrifuge capable of 264,000 g
19. Ultracentrifuge rotors such as TLA-110, TLA-120.2 or TLS-55 (Beckmann)
20. 3.2 mL, Open-Top Thickwall Polycarbonate Tube for TLA-110 rotor (Beckmann, 362305)
21. 1 mL, Open-Top Thickwall Polycarbonate Tube for TLA-120.2 or TLS-55 rotors (Beckmann, 343778)
22. Acquity UPLC class-M system (Waters)
23. Acquity UPLC BEH C4 VanGuard pre-column – 300 Å, 1.7 µm, 2.1 mm x 5 mm (Waters, 186004623) or Acquity UPLC BEH C18 VanGuard pre-column – 130 Å, 1.7 µm, 2.1 mm x 5 mm (Waters, 186003975)
24. Acquity UPLC HSS T3 Column – 100 Å, 1.8 µm, 1 mm x 50 mm (Waters, 186003535)
25. Synapt G2Si Mass Spectrometer (Waters)
26. ProteinLynx Global Server Software (Waters)
27. DynamX HDX Data Analysis Software 3.0 (Waters)
28. Pierce Disposable Columns, 2 mL (Thermo Scientific, 10066073)
29. HisTrap HP 5 mL column (Cytiva, 17524802)
30. Superdex 200 Increase 10/300 GL (Cytiva, 28990944)
31. RESOURCE Q (Cytiva, 17117901)
32. 0.22 µm, hydrophilic PVDF, ultracentrifugal filter (Merck, UFC30GVNB)
33. Zeba 7K spin column (ThermoScientific, 89882)

### Reagent Setup

**CRITICAL** Dissolve all chemicals in ultrapure water in the indicated concentrations, unless noted otherwise.

1. **TB medium –** For 1L, dissolve 12 g of tryptone, 24 g of yeast extract, 9.4 g of K_2_PO_4_ and 2.2 g of KH_2_PO_4_ in 996 mL of water. Add 4 mL of glycerol. Autoclave the medium and store it at RT, where it is stable for months.
2. **LB medium –** For 1 L, dissolve 10 g of tryptone, 5 g of yeast extract and 10 g of NaCl in water. Autoclave the medium and store it at RT, where it is stable for months.
3. **50x M** – For 100 mL, dissolve 33.5 g Na_2_HPO_4_ x 7 H_2_O, 17 g KH_2_PO_4_, 13.4 NH_4_Cl, and 3.6 g Na_2_SO_4_ in water. If crystals appear, dissolve by stirring combined with mild heating. Filter-sterilise the solution and store it at RT where it is stable for months.
4. **50x 5052 –** For 100 mL, dissolve 25 g of glycerol, 2.5 g of glucose and 10 g of α-lactose in water. Lactose is slow to dissolve and may require stirring for hours at RT. Mild heating can be used to speed up the process. Filter-sterilise the solution and store it at RT where it is stable for months.
5. **Trace metal solutions, 0.1-1 M** – Dissolve FeCl_3_, CaCl_2_, MnCl_2_ x 4 H_2_O, ZnSO_4_ x 7 H_2_O, CoCl_2_, CuCl_2_ x 2 H_2_O, NiCl_2_ x 6 H_2_O, Na_2_MoO_4_, or Na_2_SeO_3_ in water. Autoclave solutions and store them at RT where they are stable for months.
6. **1000x trace metals –** For 10 mL, mix 5 mL of 0.1 M FeCl_3_, 200 µL of 1 M CaCl_2_, 100 µL of 1 M MnCl_2_ x 4 H_2_O, 100 µL of 1 M ZnSO_4_ x 7 H_2_O, 100 µL of 0.2 M CoCl_2_, 200 µL of 0.1 M CuCl_2_x2 H_2_O, 100 µL of 0.2 M NiCl_2_ x 6 H_2_O, 200 µL of 0.1 M Na_2_MoO_4_, 200 µL of 0.1 M Na_2_SeO_3_, and 200 µL of 0.1 M H_3_BO_3_. Top up with water to 10 mL volume. Autoclave the solution and store it at RT where it is stable for months.
7. **ZYM-5052 auto-induction media –** For 1 L, dissolve 10 g of tryptone and 5 g of yeast extract in 958 mL of water. Add 2 mL of 1 M MgSO_4_, 20 mL of 50x M, 20 mL of 50x 5052 and 200 µL of 1000x trace metals. Autoclave the medium and incubate at 30 °C for 3 days to confirm sterility. Store at 4 °C where medium is stable for months.
8. **Ampicillin, 100 mg/mL –** Dissolve ampicillin sodium salt in water. Filter-sterilize the solution and store it at -20 °C. The solution is stable for months.
9. **Chloramphenicol, 34 mg/mL** – Dissolve chloramphenicol in 100% ethanol. Store solution at -20 °C. The solution is stable for months.
10. **IPTG, 1 M –** Dissolve in water. Filter-sterilize the solution and store it at −20 °C. The solution is stable for at least 1 year.
11. **Lysozyme, 50 mg/mL** – Resuspend 500 mg of lysozyme in 5 mL of PBS. Do not shake or vortex the solution. Allow resuspension to occur by gentle mixing at 4 °C. Once all lysozyme resuspends, add 5 mL of glycerol and gently mix the solution. Store solution at -20 °C where it is stable for months.
12. **Tris-HCl, 1 M** – Dissolve Tris base in water. Adjust the pH to 7.5 or 8.0 with HCl. **! CAUTION** HCl is corrosive. Wear gloves and eye protection. Filter-sterilize the solution and store it at RT. The solution is stable for months.
13. **NaCl, 5 M** – Dissolve in water. Filter-sterilize the solution and store it at RT (20-25 °C). The solution is stable for years.
14. **Imidazole, 1 M** – Dissolve in water. Adjust the pH to 8.0 with NaOH. **! CAUTION** Imidazole and NaOH are corrosive. Wear gloves and eye protection. Filter-sterilize the solution and store it at RT in the dark. The solution is stable for months.
15. **MgSO**_**4**_, **1 M** - Dissolve in water. Filter-sterilize the solution and store it at RT. The solution is stable for years.
16. **KOAc, 6 M** – Dissolve in water. Filter-sterilize the solution and store it at RT. The solution is stable for years.
17. **KCl, 1M** – Dissolve in water. Filter-sterilize the solution and store it at RT. The solution is stable for years.
18. **MgCl**_**2**_, **1 M** – Dissolve in water. Filter-sterilize the solution and store it at RT. The solution is stable for years.
19. **Mg(OAc)**_**2**_, **1 M** – Dissolve in water. Filter-sterilize the solution and store it at RT. The solution is stable for years.
20. **HEPES-NaOH, 1 M** – Dissolve in water. Adjust the pH to 7.5 with NaOH. **! CAUTION** NaOH is corrosive. Wear gloves and eye protection. Filter-sterilize the solution and store it at RT. The solution is stable for months.
21. **DTT, 1 M** – Dissolve in water. Filter-sterilize the solution and store it at -20 °C, where the solution is stable for months.
22. **RNC lysis buffer –** The buffer contains 70 mM Tris-HCl, pH 7.5, 150 mM KCl, 10 mM MgCl_2_, 8 U/mL RiboLock RNase inhibitor, and 0.5x Halt Protease Inhibitor Cocktail. Prepare 50 mL solution by adding 3.5 mL of 1 M Tris-HCl pH 7.5, 7.5 mL of 1 M KCl, 500 µL of 1 M MgCl_2_, 10 µL of 40 U/µL RiboLock RNase inhibitor and 250 µL of 100x Halt Protease Inhibitor Cocktail to 38 mL of RNase-free water. Filter-sterilize (0.22 µm) the solution and store it at −20 °C. The solution is stable for months.
23. **RNase-free DNase, 3 KU/µL** – Resuspend lyophilised RNase-free DNase corresponding to 1500 Kunitz units in 500 µL of RNase-free water. Store solution at - 20 °C where it is stable for months.
24. **RNC buffer -** The buffer contains 50 mM HEPES-NaOH, pH 7.5, 100 mM KOAc, 12 mM Mg(OAc)_2_, 1 mM DTT, and 8 U/mL RiboLock RNase inhibitor. Prepare 50 mL solution by adding 2.5 mL of 1 M HEPES-NaOH pH 7.5, 833 µL of 6 M KOAc, 600 µL of 1 M Mg(OAc)_2_, 50 µL of 1M DTT, and 10 µL of 40 U/µL RiboLock RNase to 46 mL of RNase-free water. Filter-sterilize (0.22 µm) the solution and store it at −20 °C. The solution is stable for months.
25. **Sucrose cushion buffer** – The buffer contains 35% w/v RNase-free sucrose, 50 mM HEPES-NaOH, pH 7.5, 1 M KOAc, 12 mM Mg(OAc)_2_, 1 mM DTT, 8 U/mL RiboLock RNase inhibitor, and 0.2x Halt Protease Inhibitor Cocktail. Prepare 50 mL solution by adding 17.5 g RNase-free sucrose, 2.5 mL of 1 M HEPES-NaOH pH 7.5, 8.33 mL of 6 M KOAc, 600 µL of 1 M Mg(OAc)_2_, 50 µL of 1M DTT, 10 µL of 40 U/µL RiboLock RNase, and 100 µL of 100x Halt Protease Inhibitor Cocktail to RNase-free water for a final volume of 50 mL. Filter-sterilize (0.22 µm) the solution and store it at −20 °C. The solution is stable for months.
26. **GFP-clamp resin** – Prepare according to BOX 1
27. **GFP-clamp lysis buffer** – The buffer contains 25 mM Tris, pH 8 and 150 mM NaCl.
  Prepare 1 L by adding 25 mL of 1M Tris-HCl, pH 8 and 30 mL of 5 M NaCl to 945 mL of water. Filter-sterilize the solution and store it at RT. The solution is stable for months.
28. **GFP-clamp running buffer** – The buffer contains 25 mM Tris, pH 8 and 20 mM NaCl.
  Prepare 1 L by adding 25 mL of 1M Tris-HCl, pH 8 and 4 mL of 5 M NaCl to 971 mL of water. Filter-sterilize the solution and store it at RT. The solution is stable for months.
29. **GFP-clamp regeneration buffer –** The buffer contains 8 M urea, 20 mM glycine, pH 1.5. To prepare 30 mL, dissolve 14.4 g of ultra-pure urea and 45 mg of glycine in RNase-free water. Adjust pH to 1.5 with HCl. Filter-sterilise the solution. **CRITICAL** Always make up solution fresh.
30. **PBS buffer –** The buffer contains 137 mM NaCl, 3.4 mM KCl, 10 mM Na_2_HPO_4_ and 1.8 mM KH_2_PO_4_. For 1 L dissolve 8 g NaCl, 0.25 g KCl, 1.44 g Na_2_HPO_4_ and 0.25 g KH_2_PO_4_ in water. Autoclave the medium and store it at RT, where it is stable for months.
31. **3C buffer** – The 3C buffer contains 20 mM Tris-HCl pH 8, 150 mM NaCl, 1 mM DTT and 15% v/v glycerol. To make 100 mL, add 2 mL of 1 M Tris-HCl, pH 8, 3 mL of 5 M NaCl, 100 µL of 1 M DTT and 15 mL of glycerol to 80 mL of water. Mix and filter-sterilise the solution. Store the solution at 4 °C. The solution is stable for weeks.
32. **HRV 3C protease, 5 mg/mL** – dilute purchased or home-made purified protease in 3C buffer to a final concentration of 5 mg/mL. Make 25 µL aliquots. Snap-freeze in liquid nitrogen and store at -80 °C. Protein is stable for years.
33. **Reducing sample buffer, 4×** - Add 333 µL of β-mercaptoetahnol to 10 mL of NuPAGE LDS Sample Buffer 4x.
34. **PBS-T buffer –** Buffer contains PBS with 0.05% v/v Tween-20. For 1 L, add 0.5 mL of Tween-20 to 1 L of PBS. Store at RT, where the buffer is stable for months.
35. **Blocking buffer –** Buffer contains PBS with 0.05% v/v Tween-20 and 5% w/v skimmed dry milk. For 100 mL, dissolve 5 g of skimmed dry milk in 100 mL of PBS-T buffer.
36. **Sodium citrate, 50 mM** - For 1 L, dissolve 14.7 g of trisodium citrate dihydrate in 950 mL of H_2_O. Adjust pH to 5.0 and then adjust final volume to 1 L with H_2_O. Store at 2-8 °C. The solution is stable for months.
37. **2 M Na**_**2**_**SO**_**4**_ **in 50 mM sodium citrate** – For 100 mL, dissolve 28.4 g of Na_2_SO_4_ (MW = 142.04 g/mol) in 95 mL of 50 mM sodium citrate, pH 5. Adjust pH to 5.0 and then adjust final volume to 100 mL with 50 mM sodium citrate. **CRITICAL** It is important that this solution is saturated and since 2 M Na_2_SO_4_ is above the solubility threshold, not all salt will go into solution. When using this solution, make sure all the solid has settled and only use the liquid from the top.
38. **1 M ethanolamine in 50 mM sodium citrate** – Add 6.06 mL of 16.5 M ethanolamine to 80 mL of 50 mM sodium citrate, pH 5. Adjust pH to 5.0 and then adjust final volume to 100 mL with 50 mM sodium citrate. **! CAUTION** ethanolamine is corrosive. Wear gloves and eye protection.
39. **1 M NaCl in 50 mM sodium citrate –** Dissolve 11.68 g NaCl in 190 mL of 50 mM sodium citrate, pH 5. Adjust pH to 5.0 and then adjust final volume to 200 mL with 50 mM sodium citrate.
40. **TFA, 0.08% v/v** – Add 40 µL of TFA into 50 mL of H_2_O. Final pH should be ~ 2.0.
41. **Immobilised pepsin –** Prepare according to BOX 2
42. **H-buffer** – The buffer contains 10 mM HEPES, pH 7.5, 30 mM KOAc, 12 mM Mg(OAc)_2_, and 1 mM DTT in water. For 50 mL add 250 µL of 6 M KOAc, 500 µL of 1 M HEPES-KOH, pH 7.5, 600 µL of 1 M Mg(OAc)_2_ and 50 µL of 1 M DTT to 48.6 mL of RNase-free water. Adjust pH to 7.5. Filter-sterilise the solution and store at -20 °C. The solution is stable for months.
43. **D-buffer** – The buffer contains 10 mM HEPES, pH 7.5, 30 mM KOAc, 12 mM Mg(OAc)_2_, and 1 mM DTT in heavy water (D_2_O). For 50 mL add 250 µL of 6 M KOAc, 500 µL of 1 M HEPES-KOH, pH 7.5, 600 µL of 1 M Mg(OAc)_2_ and 50 µL of 1 M DTT to 48.6 Deuterium oxide (D_2_O). Adjust pH to 7.1 with DCl or NaOD. **! CAUTION** DCl and NaOD are corrosive. Wear gloves and eye protection. Filter-sterilise the solution and store at -20 °C. The solution is stable for months.
44. **Q-buffer** – The buffer contains 100 mM NaH_2_PO_4_, pH 1.4, 10 mM TCEP-HCl, and 4 M GuHCl. For 100 mL, mix 1.38 g of NaH_2_PO_4_ x H_2_O, 287 mg of TCEP-HCl and 38.2 Guanidine-HCl. Add RNase-free water to final 90 mL volume and continue stirring until everything is dissolved. Adjust pH to 1.4 with orthophosphoric acid. Top up the buffer to final 100 mL with RNase-free water. Confirm that upon mixing the Q-buffer with the H-buffer at 1:1 ratio, the resulting pH is between 2.3 and 2.6. Further adjust the pH of the Q-buffer if necessary. Note down the exact amount of orthophosphoric acid added, so that the same buffer can be remade in the future, ensuring consistency. Filter-sterilise the solution and store it at -20 °C, where the solution is stable for months. **CRITICAL** Including GuHCl in this buffer is necessary to prevent nucleic acid precipitation at low pH ^78^.
45. **[Glu**^**1**^**]-Fibrinopeptide B, 0.25 mg/mL** – For 2 mL, dissolve 0.5 mg of [Glu^1^]-Fibrinopeptide B (GluFib) in 2 mL LC/MS-grade water. Store at -80 °C where the solution is stable for a year.
46. **Lockspray solution** – Dilute 125 µL of 0.25 mg/mL GluFib in 100 mL of 50:50 acetonitrile/water with 0.1% FA for a final 200 fmol/μl concentration. This solution is stable for months at RT.

### Equipment Set Up

1. **LC/MS instrument –** LC/MS in this protocol is conducted using a Waters Acquity UPLC class-M system upstream of the Synapt G2Si instrument in ion mobility (IM) mode, coupled to the HDX manager from Waters. The acquisition parameters are set to HDMS^E^ data-independent acquisition, positive ion electrospray mode, scan time of 1 s, sampling cone voltage of 30 V, m/z range of 50-2,000, lock mass of 785.84 m/z ([Glu^1^]-Fibrinopeptide B), capillary voltage of 3 kV, trap collision energy of 4 V, source temperature of 80 °C, desolvation temperature of 150 °C, IM wave velocity of 650 m/s, IM wave height of 40 V, and transfer collision energy ramp of 20 to 45 V. Note that these parameters are a guide only, and are typically tuned for each instrument. Different combinations of parameters can give comparable results. Prior to data acquisition, the mass spectrometer is calibrated with the lockspray solution at a flow rate of 5 µl/min. Subsequently, a sample run method is initiated and a blank sample of 0.1% FA in water is injected to ensure that there is no extensive background signal detected in the mass spectrometer.
2. **LC sample run method –** Pump A is connected to LC/MS-grade water acidified with 0.1% v/v formic acid. Pump B is connected to LC/MS-grade acetonitrile (ACN). Injected peptides are trapped (4 min, 200 µl/min) on a VanGuard Acquity UPLC BEH C4 or C18 pre-columns. Subsequently, the peptides are eluted onto a ACQUITY UPLC HSS T3 column. Both columns are kept in a cooling chamber set to 0.0 ± 0.2 °C throughout the whole data acquisition. The elution from the LC columns proceeds at 90 µl/min via several steps. The program includes an LC gradient of 3-10% ACN over 1.5 min followed by a gradient of 10-30% ACN over 23.5 min. The LC column is then washed by a 1 min gradient of 30-90% ACN followed by a 1 min gradient from 90% to 3% ACN. This is followed by three cycles of a 1 min gradient from 3% to 85% ACN followed by a 1 min gradient from 85% to 3%.
3. **LC wash run method** – Between sample injections a wash run is performed to remove any leftover peptides in the system and thus prevent carryover of peptides from a previous injection. Injected wash buffer is trapped (3 min, 200 µl/min) and the LC column is then washed by three cycles of a 2 min gradient from 3% to 95% ACN followed by a 2 min gradient from 95% to 3% ACN.

### Procedure

#### Stage 1: Generation of stalled RNCs | Timing 1 week

##### 1 Design of RNC expression vector | Timing 3-4 d

- Using standard molecular biology techniques, introduce the gene coding for the protein of interest followed by the stalling motif (WWWPRIRGPP) into an *E. coli* expression vector, downstream of a suitable N-terminal purification tag which is optionally followed by a protease cleavage site. **?TROUBLESHOOTING**

##### 2 Expression of RNCs | Timing 1-2 d

- Prepare a flask with 250 mL of ZYM-5052 auto-induction media supplemented with appropriate antibiotic. For pET21 vectors use 250 µL of 100 mg/mL of ampicillin for a final concentration of 100 µg/mL.
- Inoculate a growth culture by adding 1 colony of BL21(DE3) *E. coli* freshly transformed with a plasmid encoding the N-terminally tagged RNC construct.
- Incubate the growth culture overnight (16-18 hours) at 37 °C with shaking.
**?TROUBLESHOOTING**
- Harvest cells by centrifugation (4000 g, 30 min).
- Remove the supernatant and resuspend the pellet in 4-fold excess of ice-cold RNC lysis buffer – for 1 mL of pelleted cells add 4 mL of buffer.
- Transfer resuspended cells into a 15 mL plastic conical tube.
- Freeze cells by incubation in -80 °C or snap-freezing in liquid nitrogen.
  **PAUSE POINT** – Frozen cells can be stored at -80 °C for weeks.

#### Stage 2: Purification of stalled RNCs | Timing 2-3 days

##### 3 Lysis | Timing 2 h

- Thaw the resuspended cells by short incubation at RT.
- Add ~200 µL of 50 mg/mL lysozyme for a final concentration of ~2 mg/mL.
- Incubate cells with lysozyme for at least 30 min at 4 °C with gentle mixing.
- Freeze cells by incubation in -80 °C or snap-freezing in liquid nitrogen.
- Thaw resuspended cells by short incubation at RT. At this stage, samples should be lysed and highly viscous because of the high concentration of DNA in the solution. If the sample is not viscous, repeat the freeze-thaw cycle one or two more times.
- Add 100 µL of 3 KU/µL RNase-free DNase for a final concentration of ~0.05 Kunitz units/µL.
- Incubate cells with DNase for at least 15 min at 4 °C with gentle mixing, or until solution stops being viscous. More DNase can be added to speed up this process.
- Separate the soluble fraction by centrifugation (20 min, 16,000 g) and transfer the supernatant to a new tube kept at 4 °C. If the supernatant is not clarified completely, repeat the centrifugation.

##### 4 First sucrose cushion ultracentrifugation | Timing 5 h

- Fill thick-walled polycarbonate tubes, compatible with high-speed centrifugation and the available rotors, with ice-cold RNC sucrose buffer to half their nominal volume. For pellets from 250 mL cultures, we recommend using the Beckman TLA-110 rotor with 3.2 mL tubes filled with 1.6 mL of the RNC sucrose buffer.
- Layer the supernatant on top of the sucrose cushion to fill the polycarbonate tubes to their nominal volume. Pipette the supernatant slowly to minimise mixing of the two layers.
- Use as many polycarbonate tubes as necessary to distribute all the supernatant over a sucrose cushion.
- Ensure all tubes have the same mass. If necessary, adjust volume of the supernatant layer with RNC lysis buffer. If the supernatant is distributed across an odd number of tubes, fill an extra polycarbonate tube with RNC sucrose buffer and water for balancing.
- Carefully place the tubes into a pre-cooled (4 °C) rotor compatible with the selected polycarbonate tubes. Place the rotor into a pre-cooled (4 °C) ultracentrifuge.
- Centrifuge samples (264,000 g, 4 °C) for at least 2 hours.
- Immediately remove all supernatant, making sure to not disrupt the clear pellet.
- Carefully add 500 µL of ice-cold RNC buffer, to wash the pellet. Immediately remove all the liquid.
- One more time, carefully add 500 µL of ice-cold RNC buffer and immediately remove all the liquid.
- Add ~150 µL of ice-cold RNC buffer and make sure the pellet is covered with liquid.
- Cover open-top tubes with parafilm.
- Incubate tubes in 4 °C for 2 h with gentle rocking or until the pellet is fully resuspended. Pellets can be left resuspending up to 24 hours.
- Combine solutions containing resuspended pellets into a single RNase-free tube.

##### 5 Affinity purification – binding to resin | Timing 30 min + incubation overnight

- Prepare a purification column by inserting the frit into a 2 mL disposable column.
- Resuspend GFP-clamp agarose (BOX 1) by gentle mixing to have a homogeneous 50% slurry. Add 500 µL of the slurry to the disposable column.
- Wait for all buffer to drip through the column. Discard the buffer.
- Add 1 mL of RNC buffer to the beads and wait for the buffer to drip through the column. Discard the buffer.
- Repeat the previous step one more time.
- Put the bottom cap on the column and add resuspended pellets to the beads.
- Mix by pipetting up and down. Close the column with the top cap.
- Incubate at 4 °C overnight (16-18 hours) with gentle rotation.

##### 6 Affinity purification – elution from resin | Timing 4-5 h

- Remove top and bottom caps from the column. Discard the flow-through.
- Add 1 mL of RNC buffer to the beads and wait for the buffer to drip through the column. Discard the buffer.
- Repeat the previous step two more time.
- Put the bottom cap on the column.
- Add 225 µL of RNC buffer to the beads, then add 25 µL of 5 mg/mL HRV 3C protease. **?TROUBLESHOOTING**
- Mix by pipetting up and down and close the column with the top cap.
- Incubate at 4 °C for at least 4 hours with gentle rotation. If convenient, samples can be incubated at 4 °C for up to 1 day.
- Remove top and bottom caps from the column. Collect the elution from the column to an RNase-free LoBind tube.
- Add 250 µL of RNC buffer to the beads, mix gently by pipetting up and down, and collect all buffer from the column to the same RNase-free tube.
- Measure absorbance at 260 nm of the elution sample.
- Add 200 µL of RNC buffer to the column and immediately close both caps. Store column at 4 °C. At the end of the purification, beads can be regenerated by one wash with 1 mL of PBS, three washes with 1 mL of GFP-clamp regeneration buffer, and 2 washes with 1 mL of PBS.

##### 7 Second sucrose cushion ultracentrifugation | Timing 4-5 h

- Fill thick-walled polycarbonate tubes, compatible with high-speed centrifugation and the available rotors, with ice-cold RNC sucrose buffer to half their nominal volume. For elution from 250 mL cultures, we recommend using 1 mL tubes filled with 0.5 mL of the RNC sucrose buffer.
- Layer the elution on top of the sucrose cushion. Pipette the elution slowly to minimise mixing of the two layers. Adjust the volume in the polycarbonate tube to the nominal volume using ice-cold RNC buffer.
- Make up an extra polycarbonate tube with RNC sucrose cushion and water for balancing. Ensure both tubes have the same mass. If necessary, adjust the volumes with RNC buffer.
- Carefully place the tubes into a pre-cooled (4 °C) rotor compatible with the selected polycarbonate tubes. Place the rotor into a pre-cooled (4 °C) ultracentrifuge compatible with the rotor.
- Centrifuge samples (264,000 g, 4 °C) for at least 2 hours.
- Immediately remove all supernatant, making sure to not disrupt the clear pellet.
- Carefully add 500 µL of ice-cold RNC buffer, to wash the pellet. Immediately remove all the liquid.
- Add ~20-50 µL of ice-cold RNC buffer to ensure the pellet is just covered with liquid.
- Cover open-top tubes with parafilm.
- Incubate tubes in 4 °C for 2 h with gentle rocking or until the pellet is fully resuspended. Pellets can be left resuspending for up to 1 day.
- Collect resuspended pellets into an RNase-free LoBind tube.
- Measure absorbance of your sample at 260 nm to record the concentration of the purified RNC (100 A_260nm_ = 2.4 µM = 2.4 pmol/µL). **?TROUBLESHOOTING**
- Keep purified sample at 4 °C if intended for immediate use or snap-freeze in liquid nitrogen and store at −80 °C.

##### 8 RNC quality control | Timing 2 h

- Treat an aliquot (~4 pmol) of the purified RNC with final 50 mM EDTA and 50 µg/mL RNaseA in RNC buffer. Allow RNase to digest the RNC for 5 min at 37 °C.
- Prepare or purchase denaturing SDS-PAGE Bis-Tris gels with a polyacrylamide concentration that permits resolution of the nascent chain of interest. Typically, 4-12% gels are appropriate to resolve NCs of at least 100 amino acids. 10% or 12% gels are appropriate to resolve shorter NCs.
- Load the gel with ~4 pmol of empty 70S ribosomes, purified untreated RNC, and RNC treated with RNaseA. Use reducing sample buffer at final 1x dilution as the loading dye.
- Run the SDS-PAGE in 1x MES SDS running buffer for 50 min at 180 V and subsequently stain resolved protein bands with Coomassie stain.
- Investigate the stained gel for confirming sample purity and integrity. High-quality RNC samples should contain the same bands at similar intensity as seen in the empty 70S control. **?TROUBLESHOOTING**
- Additionally, RNC samples should contain an additional RNase-sensitive band with the apparent MW about 20 kDa larger than the theoretical MW of the NC, due to the presence of the tRNA. Upon RNase treatment, the band should drop by ~20 kDa due to tRNA degradation. **?TROUBLESHOOTING**
- Optional: Confirm that the intensity of the NC band is comparable to intensity of 4 pmol of isolated protein of interest corresponding to the NC to validate the NC occupancy of the ribosome. **?TROUBLESHOOTING**
- Optional: Confirm sample composition by mass spectrometry analysis. **PAUSE POINT** Purified RNCs are stable at -80 °C for months.

#### Stage 3: Sample preparation for HDX-MS | Timing 1-2 days

##### 9 Preparation of protein stocks | Timing 1 h

- Measure the concentration of the purified RNC (100 A_260_ = 2.4 µM). If necessary, dilute samples to ~5 µM in RNC buffer. Place sample on ice.
- Dilute empty 70S ribosomes to the same concentration as the RNC in RNC buffer. Place sample on ice.
- Dilute isolated proteins of interest (such as the native protein corresponding to the NC or any RNC-binding proteins present in the RNC sample) to the same concentration as the RNC in RNC buffer. Place samples on ice.

##### 10 Preparation of undeuterated samples | Timing 15 min per sample

- Equilibrate 1 mL of H-buffer to 25 °C in a thermocycler set to 350 rpm shaking.
- Place a 0.22 µm PVDF ultracentrifugal filter in a collection tube in a 10 °C thermocycler shaking at 450 rpm.
- Transfer 20 µL of 50% pepsin-coupled bead slurry in 0.1% FA (BOX 2) into the filter.
- For samples at 1-5 µM concentration, mix 3 µL of sample with 27 µL H-buffer at 25 °C. For samples at concentrations below 1 µM, buffer-exchange 30 µL of sample into H-buffer using 0.5 mL Zeba 7K spin columns (1000 g, 25 °C, 2 min) pre-equilibrated with 4 × 400 µL H-buffer.
- Mix 30 µL of samples in H-buffer with 30 µL of ice-cold Q-buffer.
- Immediately take up the total volume (60 µL) of the quenched sample and transfer it to the filter containing pepsin-coupled beads.
- Incubate sample with pepsin-coupled beads in the filter for 100 seconds (10 °C, 450 rpm). Quickly vortex the tube containing the filter every 30 s.
- Spin the collection tube containing the filter for 15 s at 13,000 g at 0 °C.
- Following the spin, remove the filter with beads and immediately snap-freeze the flow-through sample in liquid nitrogen.
- Repeat from the first step to make undeuterated samples in three replicates. Samples can be stored in -80 °C where they are stable for weeks.

##### 11 Preparation of deuterated samples | Timing 1-2 h per sample

- **CRITICAL** HDX-MS is very sensitive to changes in pH, temperature and buffer conditions. Hence, it is necessary to keep temperatures, buffers and volumes consistent throughout sample preparation.
- **CRITICAL** To ensure high-quality results it is necessary to strictly follow the exact timings of each step during preparation of deuterated samples.
- Equilibrate 1 mL of D-buffer to 25 °C in a thermocycler set to 350 rpm shaking.
- Place a 0.22 µm PVDF ultracentrifugal filter in a collection tube in a 10 °C thermocycler shaking at 450 rpm.
- Incubate sample stocks at 25 °C for 2 min.
- For samples at 1-5 µM concentration, mix 3 µL of sample with 27 µL D-buffer at 25 °C. For samples at concentrations below 1 µM, buffer-exchange 30 µL of sample into D-buffer using 0.5 mL Zeba 7K spin columns (1000 g, 25 °C, 2 min) pre-equilibrated with 4 × 400 µL D-buffer at 25 °C.
- Incubate labelling reaction at 25 °C with 350 rpm shaking. Incubation time can vary, but we recommend starting with ~10 s, ~100 s and ~1000 s time points. Note that for samples below 1 µM, the time of buffer exchange is part of the incubation, hence for those samples ~150 s is the shortest possible time point.
- Prior to labelling (for 10 s labelling time) or just before the end of the labelling period (for longer labelling times), transfer 20 µL of 50% pepsin-coupled bead slurry in 0.1% FA into the filter at 10 °C.
- After the selected labelling incubation time has passed, mix 30 µL of labelled samples in D-buffer with 30 µL of ice-cold Q-buffer.
- Immediately take up the total volume (60 µL) of the quenched sample and transfer it to the filter containing pepsin-coupled beads.
- Immediately take up the total volume of the quenched sample and transfer it to the filter containing pepsin-coupled beads. **CRITICAL** Once samples have been quenched, it is important to work as fast as possible to minimise back-exchange of deuterium label back to hydrogens from the solution.
- Incubate sample with pepsin-coupled beads in the filter for 100 seconds (10 °C, 450 rpm). Quickly vortex the tube containing the filter every 30 s.
- After the incubation with pepsin has finished, spin the collection tube containing the filter for 15 s at 13,000 g at 0 °C.
- Following the spin, remove the filter with beads and snap-freeze the flow-through sample in liquid nitrogen.
- Make each deuterated sample in three replicates at each labelling time point. **PAUSE POINT** Samples can be stored in -80 °C where they are stable for 1-2 weeks.

#### Stage 4: HDX-MS data acquisition | Timing 1-2 weeks

##### 12 Data collection | Timing 1 hour per injection

- Start the sample run method. **CRITICAL** To minimise back-exchange make sure that the liquid chromatography cooling chamber is equilibrated at 0.0 ± 0.2 °C before each run, and work with samples as fast as possible.
- Thaw an HDX sample and immediately inject it into the HDX sample manager housing a 50 µL sample loop, and initiate the LC/MS sample run.
- Wait for the run to reach the gradient phase and then inject 3 × 100 µL of 0.1% FA in water to wash the sample loop and tubing.
- After the sample run finishes (~40 min), initiate the wash run method.
- Inject 100 µL of 0.1% FA in water to initiate the wash run (~15 min).
- Optional: Start the sample run method and inject a blank sample of 0.1% FA. Ensure that there is no extensive background signal detected in the mass spectrometer. **?TROUBLESHOOTING**

**PAUSE POINT**

#### Stage 5: HDX-MS data analysis | Timing 2-8 weeks

##### 13 Obtaining a peptide list from undeuterated tandem MS data | Timing 1-2 h

vii. Create a FASTA file containing the protein sequences of porcine pepsin, all 70S ribosomal proteins, the nascent chain and any other proteins present in the injected samples such as RNC-specific interactors or major contaminants.
viii. In ProteinLynx Global Server (PLGS) software, create a new workflow parameter template with an electrospray MS^E^ search type, the FASTA file as the databank, primary digest reagent set to non-specific, missed cleavages set to 0, no fixed modifier reagent, variable modifier reagents set to Oxidation M, FDR of 4, keeping all other parameters default. Note that other combinations of parameters can be used, depending on sample quality and complexity.
ix. Create a new processing parameter template with an electrospray MS^E^ acquisition type. Set the lock mass to 785.842 Da/e (GluFib). Leave other parameters as default (low energy threshold of 135, elevated energy threshold of 30, lock mass window of 0.25 Da). Note that other combinations of parameters can be used, depending on sample quality and complexity as well as instrument type used.
x. Process raw data from all undeuterated samples with the personalised workflow and processing parameters.

##### 14 Search for peptides in raw MS^1^ data | Timing 1-2 days

- In DynamX software, import PLGS results. Filter peptides based on the proteins of interest, minimum products per amino acid of 0.05, minimum consecutive products of 1 and optionally maximum peptide length of 35. Note that other combinations of parameters can be used, depending on sample quality and complexity.
- Upload raw MS data of all collected samples – RNCs, 70S ribosomes (negative control) and isolated proteins (positive control), sort files by state (sample type) and exposure (labelling) time.
- Perform the automated ion search and peptide assignment. **CRITICAL** With such complex samples, containing over 50 ribosomal proteins, it is not possible to rely on the automatic assignments. The automatic assignment functions only as a starting point for downstream manual curation of peptide assignments.

##### 15 Removal of falsely assigned peptides | Timing 1-5 days

- Go through the automatically assigned peptides individually.
- Unassign and exclude any peptides that were automatically assigned to non-ribosomal proteins but were also found in the 70S ribosomal control.
- Optionally, unassign and exclude any peptides that were automatically assigned to a non-ribosomal protein of interest (such as the NC or RNC-interactors) in the RNC samples but not in the reference samples containing only the isolated proteins of interest. This step adds extra confidence to the peptide assignment.

##### 16 Manual inspection of assigned peptides | Timing 1-6 weeks

- Go through the automatically assigned peptides individually.
- For each peptide, ensure that a complete single isotopic envelope has been assigned. If the assignment cannot be corrected and is ambiguous because of low signal or multiple overlapping spectra, unassign and exclude the peptide.
- For each peptide, ensure that the theoretical mass of the peptide corresponds to the experimentally determined mass. If masses do not match, unassign and exclude the peptide.
- For each peptide, ensure that the ion mobility drift times are similar (within 2), across the peptide envelope in each sample and across all assigned samples. If not consider unassigning and excluding the peptide.
- For each peptide ensure that the retention time is similar (within 0.5) across the peptide envelope in each sample and across all assigned samples. If not, consider unassigning and excluding the peptide.
- For each peptide, ensure that only the most confidently assigned charge state of the peptide is retained.
- Confirm that overlapping peptides exhibit similar uptakes. Check the assignment of any outliers.
- Optional: Once the final peptide list is confirmed, it can be practical to save a separate version of the file where you delete all the excluded peptides and purge the database to make DynamX files smaller and faster to work with.

##### 17 Exporting informative values | Timing 1-3 h

- To obtain information about the conformational state of individual proteins of interest, calculate and export the fractional uptake of a given protein and map the uptake onto the protein sequence or structure. Fractional uptake is calculated by dividing the increase in centroid peptide mass upon deuteration by the theoretical uptake maximum for each peptide, typically equal to n-1, where n is the peptide length excluding prolines.
- To obtain information about the extent of protein folding on the ribosome, export the peptide-specific difference in relative D-uptake in the NC compared to natively folded full-length protein. Relative deuterium uptake in Da is calculated by subtracting the centroid mass of undeuterated peptides from those of deuterated peptides
- To obtain information about the potential RNC-binding sites in RNC interactors, export the peptide-specific difference in relative D-uptake in the RNC samples containing the interactors and the isolated interactors.
- To obtain information about the interactions between the NC and the ribosome, export the peptide-specific difference in relative D-uptake in the ribosomal proteins in RNCs and empty 70S ribosomes.

**?TROUBLESHOOTING**

### Timing

- Stage 1: Generation of stalled RNCs | 1 week
  ○ Step 1: Design of RNC expression vector | 3-4 d
  ○ Step 2: Expression of RNCs | 1-2 d
- Stage 2: Purification of stalled RNCs | 2-3 days
  ○ Step 3: Lysis | 2 h
  ○ Step 4: First sucrose cushion ultracentrifugation | 5 h
  ○ Step 5: Affinity purification – binding to resin | 30 min + incubation O/N
  ○ Step 6: Affinity purification – elution from resin | 4-5 h
  ○ Step 7: Second sucrose cushion ultracentrifugation | 4-5 h
  ○ Step 8: RNC quality control | 2 h
- Stage 3: Sample preparation for HDX-MS | 1-2 days
  ○ Step 9: Preparation of protein stocks | 1 h
  ○ Step 10: Preparation of undeuterated samples | 15 min per sample
  ○ Step 11: Preparation of deuterated samples | 1-2 h per sample
- Stage 4: HDX-MS data acquisition | 1-2 weeks
  ○ Step 12: Data collection | 1 h per injection
  ○ Stage 5: HDX-MS data analysis | 2-8 weeks
  ○ Step 13: Obtaining a peptide list from undeuterated tandem MS data | 1-2 h
  ○ Step 14: Search for peptides in raw MS^1^ data | 1-2 days
  ○ Step 15: Removal of falsely assigned peptides | 1-5 days
  ○ Step 16: Manual inspection of assigned peptides | 1-6 weeks
  ○ Step 17: Exporting informative values | 1-3 h

### Troubleshooting

**Table:** 

Step	Problem	Possible reason	Solution
2 iii.	Protein cannot be expressed at 37 °C.	NC or its binding partner is not stable at 37 °C.	Expression can also be achieved at lower temperatures for longer. We have had success with incubating ZYM-5052 cultures at 37 °C for 5 hours followed by incubation at 18 °C for 48 hours. Alternatively, cultures can be set up in LB or TB media, grown at 37 °C until OD_600_ reaches 0.6-0.8, induced with 1 mM IPTG and incubated for expression at desirable conditions.
1 i. or 6 v.	The muGFP tag cannot be used for NC purification.	The muGFP tag interferes with NC expression, folding or interactions. OR The tag cannot be cleaved during purification as it needs to be kept on the RNC for downstream analysis.	Change the N-terminal tag. We have had success with an N-terminal 3xFLAG tag or an N-terminal Twin-Strep tag. We do not recommend using a His_6x_ tag. 3xFLAG peptide can be used to elute 3xFLAG tag. Biotin or d-desthiobiotin can be used to elute Twin-Strep tag.
7 xiii.	The RNC purification yield is low.	Poor expression, poor binding to affinity resin, poor elution, tag-degradation, RNase contamination.	Collect small aliquots throughout expression and purification steps and use SDS-PAGE followed by detection of muGFP fluorescence, Coomassie staining or immunoblotting to identify at which step yields are compromised.
8 v.	The RNC sample is impure.	Contamination during the ultracentrifugation steps.	Make sure the sucrose cushion is always at least 0.5 of the nominal volume of the tube. Make sure the cushion is cold when loading the sample. Make sure sample is loaded slowly to not mix the sample with the sucrose cushion layer. Make sure all supernatant is removed after the sucrose cushion step and the pellet is washed twice.
8 v.	The purified RNC did not co-purify with high levels of a known interactor (e.g. Trigger factor).	Interaction is disrupted during the purification process.	Consider lowering the salt concentration in the sucrose cushion buffer to preserve salt-sensitive interactions.
8 vi.	No RNase-sensitive band is visible upon Coomassie staining.	Small RNC bands (NC shorter than 100 residues) might be obscured by the ribosomal proteins.	We recommend performing an immunoblot with NC-specific antibodies.
8 vii.	The NC is present at sub 1:1 stoichiometry compared to the ribosomal proteins.	Contamination with empty ribosomes.	Increase the number of resin washes before the tag-cleavage step.
12 vii.	Detection of large chromatogram signal when running a blank following an HDX- MS sample and wash runs.	Peptides from the sample run were not washed efficiently from the tubing and/or LC columns.	Run two wash runs after each sample run or try to use a harsher wash solution made up of 1.5 M guanidinium-HCl, 4% v/v acetonitrile and 0.8% v/v formic acid.
17	Protein sequence is covered poorly by detected peptides.	Insufficient cleavage of the protein. This could be due to insufficient unfolding or aggregation of the sample, pepsin losing activity, or pepsin not cleaving the specific substrate sequence well.	Run SDS-PAGE of undigested and digested samples to troubleshoot why digestion is not efficient. Consider changing the quench buffer to include MS-compatible detergents, urea, higher concentrations of TCEP, or protein-stabilising components. Consider using alternative acid-stable proteases ^79^.
17	There is insufficient deuterium uptake in the majority of peptides.	Insufficient labelling or high levels of back-exchange.	Label proteins at increased temperature of for longer times. Ensure that the pH of the sample following quenching is maintained at ~2.5 throughout pepsin digestion. Prepare and analyse a maximally deuterated sample ^80^ or alternatively highly deuterated control peptides (e.g. phosphorylase B tryptic digest) to estimate the levels of back- exchange.

### Anticipated results

We have used this protocol to purify various RNCs to explore the cotranslational folding of a short, single-domain protein DHFR (159 residues, 18 kDa) ^25^ and a large, multi-domain protein β-galactosidase (1023 residues, 116 kDa) ^24,54^ as well as their interaction with molecular chaperones.

**Figure 3:**
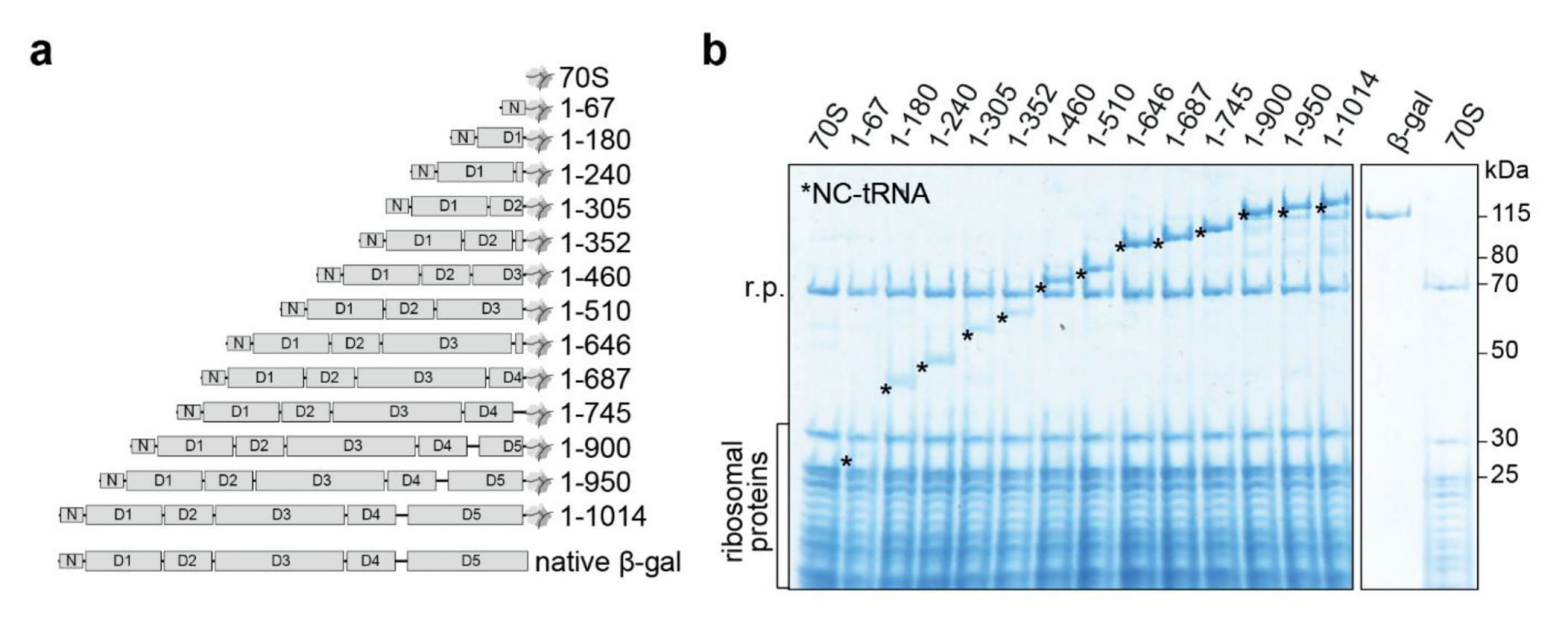
Set of purified RNCs sampling β-galactosidase synthesis (**a**) Schematic of truncated β-gal constructs encoded upstream of the SecMStr ribosome stalling sequence (WWWPRIRGPP). N-terminal extension (N) and individual domains (D1-D5) of β-gal are indicated. The empty 70S ribosomes and full-length native β-gal are included for reference. (**b**) Coomassie-stained SDS-PAGE gels of β-gal RNCs purified from TF knock-out *E. coli* cells (left) and full-length native β-gal (right). Bands corresponding to tRNA-linked nascent chains (*NC-tRNA) migrate higher, by ~20 kDa, than expected from NC molecular weight. A lane with empty 70S ribosome is included on both gels for reference to identify bands corresponding to ribosomal proteins (r.p.). This figure was adapted from a doctoral thesis ^81^.

After overnight expression of RNCs (steps 1-2), lysis (step 3), and isolation of the ribosomal fraction using sucrose cushion ultracentrifugation (step 4), we typically obtain ~2-4 nmol of ribosomes per 250 mL of culture based on absorbance at 260 nm (A_260_) of the resuspended pellet. The yield is mostly limited by the incomplete lysis of cells using the gentle freeze-thaw approach. The majority of the ribosomal content (based on A_260_) is lost during affinity purification (steps 5-6) in the flow-through and washes, which removes empty ribosomes as well as some NC-occupied ribosomes. A typical elution from the muGFP-clamp resin (step 6) contained about ~0.5-1 nmol of ribosomes (based on A_260_) per 250 mL of culture, which reduced further to ~0.25-0.75 nmol of highly purified RNCs following the second sucrose cushion ultracentrifugation (step 7). This amount was typically enough to conduct subsequent quality control (step 8, Fig 2) as well as HDX-MS experiments (steps 9-17). Note that the exact yield of the purification is affected by the specific NC being purified. If necessary, the purification can be easily scaled up by starting from a larger volume of culture. Multiple purifications (typically 5-10) can reasonably be conducted in parallel (Fig 3). Successfully purified RNCs contain all ribosomal proteins as well as the NC at a close to 1:1 occupancy, which we initially verified by quantitative mass spectrometry ^24,25^ and later confirmed with SDS-PAGE followed by Coomassie staining or immunoblotting.

**Figure 4:**
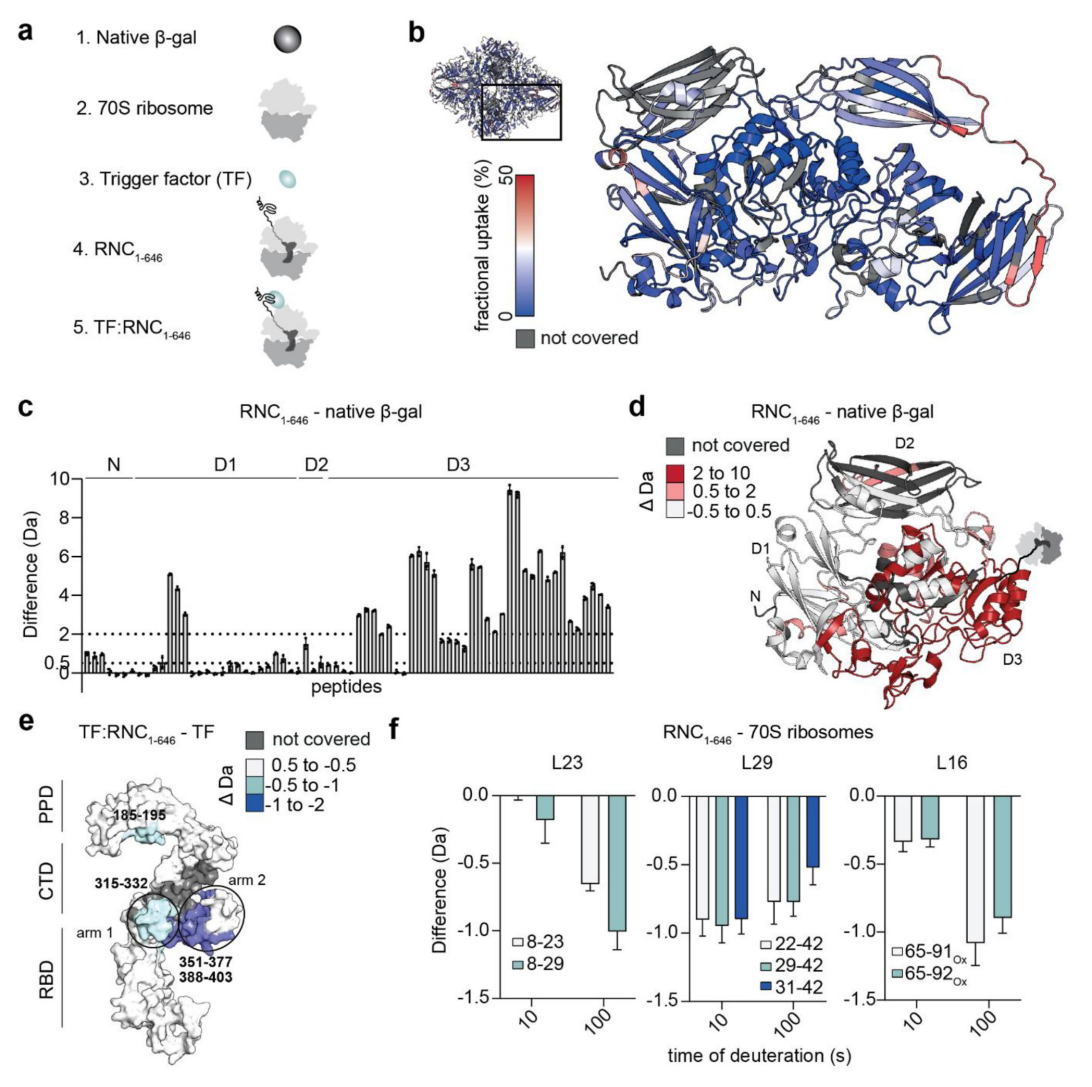
HDX-MS analysis of β-galactosidase RNC_1-646_ (**a**) Schematic of analysed samples – native full-length β-gal (1.), empty 70S ribosomes (2.), molecular chaperone Trigger factor (TF, 3.), β-gal RNC_1-646_ with ~626 residues exposed outside of the exit tunnel (4.) and a TF-bound RNC_1-646_ (5.). (**b**) β-gal tetramer (left) and monomer (right) structures (PDB: 6CVM) coloured according to the fractional deuterium uptake of native β-gal after a 10 s D_2_O pulse at 25 °C. Structure is coloured from blue to white to red for low to high uptake. Grey regions were not covered by any peptides. Plotted values are averaged over three technical replicates. *Adapted from a doctoral thesis* ^81^. (**c**) Difference in peptide-specific deuterium uptake, after a 100 s D_2_O pulse at 25 °C, between RNC_1-646_ and native β-gal. Higher values indicate more deuteration in NC_1-646_ relative to native β-gal. Error bars represent the SD of values from three independent HDX-MS datasets collected on the products of three independent RNC purifications. Each value contributing to the plotted mean is itself a mean across 2-3 technical replicates. *Adapted from a doctoral thesis* ^81^. (**d**) β-gal monomer structure (PDB: 6CVM, residues 1-626) coloured according to the relative deuterium uptake difference between RNC_1-646_ and native β-gal as shown in (c). *Adapted from published literature* ^24^. (**e**) TF structures (PDB: 1W26) coloured according to the difference in deuterium uptake (after 10 or 100 s deuteration) between TF bound to RNC_1-646_ and isolated TF. Darker blue indicates less deuteration in RNC-bound TF compared isolated TF. *Adapted from published literature* ^24^. (**f**) Deuterium uptake plots for peptides 8-23 and 8-29 in L23 (left), 22-42, 29-42 and 31-42 n L29 (middle), and methionine oxidised 65-91 and 65-92 in L16 (right). The mean difference in relative deuterium uptake between RNC_1-646_ and 70S ribosomes is plotted. Lower values indicate less deuterium uptake in the RNC. Error-bars correspond to SD of 3 technical replicates. *Adapted from a doctoral thesis* ^81^.

To illustrate how HDX-MS can be used to study cotranslational folding and chaperone interactions, we compared the deuterium uptake in native β-galactosidase, empty 70S ribosomes, molecular chaperone Trigger factor (TF), β-gal RNC_1-646_ and β-gal RNC_1-646_ bound by TF (Fig. 4a).

Following the sample preparation (steps 9-11) and data acquisition (step 12), a PLGS search based on tandem MS data of undeuterated native β-gal and RNCs (step 13) that was automatically filtered in DynamX (step 14) yielded a list of NC-covering peptides that had to be manually inspected. By removing the poor-quality peptides or those that were incorrectly or ambiguously assigned (steps 15-16), about two thirds of the peptides were excluded from analysis, highlighting the need for manual inspection. This number can vary significantly based on the specific protein of interest and stringency of the initial search parameters.

To confirm the correct assignment of the peptides in the final peptide list, we mapped the fractional D-uptake of peptides derived from native β-gal onto its solved 3D structure (step 17 i). The β-strands and alpha helices of the protein exhibited no or low levels of exchange and loops and unstructured regions exhibited higher D-uptake (Fig. 4b). This was consistent with the fact that structured regions are involved in stable hydrogen bonding and thus undergo H/D exchange at slower rates than flexible and solvent-exposed regions.

Next, plotting the difference in relative deuterium uptake in NC_1-646_ and native β-gal at peptide resolution (step 17ii), allowed us to probe the cotranslational folding of β-gal after 3 of its 5 domains have been synthesised (Fig. 4c,d), showing that while the core of domain 1 and domain 2 is folded at this stage of translation, domain 3 closest to the ribosome is unfolded. Additionally, plotting the difference in deuterium uptake between TF bound to RNC_1-646_ and isolated TF, allowed us to map the exact NC-binding sites in the molecular chaperones (Fig. 4e). Lastly, we confirmed that the presence of the NC is reflected in the uptake of specific ribosomal proteins in RNCs when compared to empty 70S ribosomes (Fig. 4f). In the presence NC_1-646_, we detected a protection from deuterium uptake in two loops - uL23_8-23_ and uL29_31-42_, positioned at the ribosome exit port where the NC emerges from the ribosome. Additionally, in the presence of the NC we detected protection of a loop, uL16_74_-_88_ near the P-site tRNA binding site, which is occupied in RNCs but not the empty 70S ribosomes.

In summary, we have shown how HDX-MS analysis of high-quality RNC samples and appropriate control samples, can be used to gain information about the structure, conformational dynamics and chaperone interactions of various nascent chains during protein synthesis on the ribosome.

## Data Availability

The mass spectrometry data have been described previously ^24^ and deposited to the ProteomeXchange Consortium via the PRIDE partner repository ^82^ with the PXD048642 dataset identifier.

## Box 1 Generating a GFP-clamp resin

### 1 Expression of GFP-clamp protein

- Prepare a starter culture by supplementing 5 mL of Terrific Broth medium (TB) with 100 µg/mL ampicillin, 34 µg/mL chloramphenicol and 1% (v/v) glucose.
- Inoculate the starter culture by adding 1 colony of Rosetta2(DE3) pLysS *E. coli* freshly transformed with a plasmid encoding the His-tagged KKK_nl_gc_R7 GFP-clamp variant described previously ^71^.
- Incubate the starter culture overnight (16-18 hours) at 37 °C with shaking.
- Inoculate a growth culture by adding the starter culture to 500 mL TB with 100 µg/mL ampicillin, 34 µg/mL chloramphenicol and 1% (v/v) glucose.
- Incubate the growth culture at 37 °C with shaking, measuring absorbance at 600 nm (OD_600_) regularly.
- When OD_600_ reaches 0.6-0.8, induce expression by adding 0.5 mL of 1 M IPTG for final concentration of 1 mM IPTG.
- Incubate the growth culture for 4 hours at 37 °C with shaking.
- Harvest cells by centrifugation (4,000 g, 20 min).
- Remove the supernatant and freeze the pellet at -80 °C until use.

### 2 Lysis of cells overexpressing GFP-clamp protein

- Thaw the bacterial pellet and resuspend cells in 50 mL of ice-cold GFP-clamp lysis buffer.
- Add lysozyme to final concentration of 1 mg/mL and 1 protease-inhibitor tablet.
- Incubate at 4 °C for 60 mins with gentle mixing.
- Lyse cells by sonication on ice – use 4 cycles of 1 min sonication (with 1 sec on, 1 sec off) and 1 min wait with 40% amplitude.
- Pellet insoluble material by centrifugation (60,000 g, 30 min, 4 °C) and isolate the supernatant.
- Filter the supernatant through 0.45 µm PVDF filter and keep at 4 °C.

### 3 Affinity purification of GFP-clamp protein

- Equilibrate a 5 mL HisTrap column with GFP-clamp lysis buffer.
- Load filtered supernatant onto the 5 mL HisTrap column at 4 °C.
- Wash column with ice-cold 15 mL of GFP-clamp running buffer supplemented with 15 mM imidazole.
- Elute GFP-clamp with ice-cold 25 mL of GFP-clamp running buffer supplemented with 300 mM imidazole. Collect elution into 1 mL fractions.
- Use SDS-PAGE to identify which elution fractions contain GFP-clamp (MW ~36 kDa) and combine these fractions.
- Add 3C protease to the pooled fractions at a ~1:50 3C:GFP-clamp ratio.
- Incubate overnight at 4 °C with gentle mixing.
- Dilute sample to 50 mL with ice-cold GFP-clamp running buffer.
- Load sample onto a RESOURCE Q column at 4 °C.
- Wash column with 30 mL of ice-cold GFP-clamp running buffer.
- Elute GFP-clamp with a 30 mL linear gradient of 25 mM to 1 M NaCl in running buffer at 4 °C. Collect elution into 1 mL fractions.
- Use SDS-PAGE to identify which elution fractions contain cleaved GFP-clamp (MW ~32 kDa).
- Concentrate pooled GFP-clamp containing fractions to ~0.5 mL.
- Load pooled fractions onto a pre-equilibrated Superdex200 (10/300) Increase column at a flow-rate of 0.5 mL/min in PBS. Collect elution into 0.5 mL fractions.
- Use SDS-PAGE to identify which elution fractions contain pure cleaved GFP-clamp (MW~32 kDa). Combine these fractions and keep protein at 4 °C.

### 4 Coupling GFP-clamp to agarose resin

- Allow Pierce NHS-Activated Agarose Slurry to settle and remove the supernatant.
- Resuspend the agarose resin in PBS to form a 50% slurry.
- Repeat the previous two steps three more times.
- Add 3 mg of purified GFP-clamp in PBS to 1 mL of bed volume of resin.
- Leave overnight at 4 °C with gentle mixing to ensure efficient coupling.
- Allow the agarose slurry to settle and remove the supernatant.
- Resuspend the agarose resin in PBS to form a 50% slurry.
- Allow the agarose slurry to settle down and remove the supernatant.
- Quench unreacted NHS groups by adding 1 mL of 1 M Tris pH 8.0 to the resin.
- Leave resin overnight 4 °C or for 2-3 hours at RT to incubate.
- Allow the agarose slurry to settle and remove the supernatant.
- Resuspend the agarose resin in PBS to form a 50% slurry.
- Keep resin at 4 °C in PBS. The muGFP-clamp resin is stable for months.

## Box 2 Preparation of immobilised pepsin

- **CRITICAL** Low pH is very important for the activity of pepsin, so be sure that pH of all solutions is measured on the day of use.
- **CRITICAL** For best yields, the preparation and use of the reducing agent as well as the coupling reactions should be conducted under an inert atmosphere such as an argon stream.
- **! CAUTION** The preparation and use of the reducing agent as well as the coupling reactions should be conducted in a chemical fume hood.

### 1 Preparation of pepsin solution

- For about 5 mL of 50% immobilised pepsin slurry, weigh 80 mg of porcine pepsin into a 100 mL round bottom flask.
- Add 2 mL of 50 mM trisodium citrate, pH 5 to the round bottom flask.

### 2 Preparation of the reducing agent

- Weigh out 20 mg of sodium cyanoborohydride into a glass vial. **! CAUTION** sodium cyanoborohydride is flammable, corrosive and toxic. Wear gloves and eye protection. Work in a chemical fume hood.
- Use syringe with a needle to add 1 mL of 2 M Na_2_SO_4_ in 50 mM sodium citrate, pH 5 to the vial.
- Mix gently by shaking at RT until sodium cyanoborohydride dissolves to yield the reducing agent.

### 3 Addition of the reducing agent to the pepsin solution

- Use syringe with a needle to take up the dissolved reducing agent.
- Inject 1 mL of the reducing agent into the round bottom flask containing dissolved pepsin.
- Mix gently by shaking for 10 minutes at RT.
- Quench any spatulas, needles or syringes that have come into contact with the reducing agent with 5% v/v ethanol.

### 4 Coupling pepsin to POROS AL-20 beads

- Add 600 mg of POROS AL-20 beads into the round bottom flask containing dissolved pepsin with a reducing agent.
- Add a stirrer bar and mix at RT.
- Wait 10 mins while solution is being stirred.
- To perform the salting out procedure, using a syringe, add 500 µL of 2M Na_2_SO_4_ in 50 mM sodium citrate, pH 5 dropwise into the round bottom flask.
- Wait 5 mins while solution is being stirred.
- Repeat the previous two steps until total of 2.2 mL of 2 M Na_2_SO_4_ in 50 mM sodium citrate, pH 5 has been added to the round bottom flask.
- Leave the coupling reaction stir overnight (12-16 h) at RT.
- Quench the coupling reaction by dropwise addition of 1 mL 1 M ethanolamine in 50 mM sodium citrate.
- Wait 2 hours, while the solution is being stirred at RT.

### 5 Isolating and cleaning pepsin-coupled beads

- Use gentle vacuum filtration to isolate coupled pepsin POROS beads.
- Wash beads with 50 mL of 50 mM sodium citrate, pH 5.0.
- Wash beads with 25 mL of 1 M NaCl in 50 mM sodium citrate, pH 5.0.
- Wash beads with 50 mL of 50 mM sodium citrate, pH 5.0.
- Wash beads with 25 mL of 0.08 % TFA, pH 2.0 in H_2_O.
- Resuspend beads as a 50% slurry in 0.08% TFA, pH 2.0 in H_2_O and transfer the slurry into a conical plastic tube.
- Sediment beads using centrifugation (7000 g, 5 min, 4 °C).
- Remove as much liquid as possible from the tube without removing beads.
- Resuspend remaining beads as a 50% slurry in 0.08% TFA, pH 2.0 in H_2_O.
- Repeat the last three steps two more times.
- Store beads as a 50% slurry in 0.08% TFA, pH 2 at 4 °C. The immobilised pepsin is stable for months.
- Just before use, take 1 mL of the 50% bead slurry and sediment beads by centrifugation (2 min, 1000 g). Remove all liquid and floating beads without removing any sedimented beads. Resuspend remaining beads in 500 µL of LC/MS-grade 0.1% formic acid in water (0.1% FA). Repeat the centrifugation and resuspension 2 more times and place the final 50% slurry of pepsin-coupled beads in 0.1% FA on ice ready for use.
